# Bad Midwifery

**Published:** 1851-08

**Authors:** 


BAD MIDWIFERY.

Dr. A. P. Stewart, of Vienna, Illinois, has communicated to us the particulars of a case in which laceration of the perineum and of the vagina occurred in the hands of an ignorant midwife. The case is by no means a rare one. Such practice as Dr. Stewart describes is much too common in many parts of our country. The natural modesty of he sex leads to a preference of female midwives, most of whom are sadly deficient in the knowledge necessary to pursue this branch of the healing art with safety, and the consequences are often most disastrous both to mother and child. In this case, the woman was shockingly mutilated, but by the judicious interference of Dr. Stewart and Dr. Shumard she was restored to health, a perfect union of the parts having been established. Under the operation of cases such as this, the prejudice against male accoucheurs must gradually give way, and the innovation will be a happy one for those who are exposed to the perils of child-bearing. Y.

